# A simple planning tool for tibial slope osteotomy—Osteotomy depth is a precise parameter to determine wedge height

**DOI:** 10.1002/ksa.70498

**Published:** 2026-06-22

**Authors:** Romed Peter Vieider, Julius Maria Watrinet, Robert Bilodeau, Sahil Dadoo, Luilly Vargas, Luke Thomas Mattar, Mahmut Enes Kayaalp, Johnathan Daniel Hughes, Volker Musahl

**Affiliations:** ^1^ Department of Orthopaedic Surgery, UPMC Freddie Fu Sports Medicine Center University of Pittsburgh Pittsburgh Pennsylvania USA; ^2^ Department of Sports Orthopaedics, TUM University Hospital Technical University of Munich Munich Germany; ^3^ Department of Orthopaedic Surgery University of Pittsburgh Pittsburgh Pennsylvania USA; ^4^ Department of Orthopaedics and Traumatology University of Health Sciences Istanbul Türkiye

**Keywords:** ACL, posterior tibial slope, slope‐reducing osteotomy, wedge height ratio

## Abstract

**Purpose:**

To compare the planning precision of three wedge height planning methods relative to angular‐based reference planning for infratubercle anterior closing wedge high tibial osteotomy (ACW‐HTO).

**Methods:**

Lateral knee radiographs of patients with posterior tibial slope (PTS) ≥ 13° were retrospectively reviewed. Infratubercle ACW‐HTO was planned targeting a PTS of 5°. Angle‐based planning served as the reference. Three methods were evaluated: wedge height calculated from osteotomy depth and correction angle (osteotomy depth method), a fixed ratio of 1.2 mm per degree of correction (ratio 1), and 1.67 mm per degree (ratio 2). Correction error was defined as the difference in wedge heights between each method. Planning precision was assessed using a ±1° correction error threshold. Intraclass correlation coefficient (ICC) was assessed.

**Results:**

Forty‐six lateral knee radiographs of 46 patients (mean age 30 ± 11 years; 44% female) were included. Mean PTS was 15° ± 2° (range: 13–19°), requiring a mean correction of 10° ± 2° (range: 8–14°). Wedge heights differed significantly across planning methods (*p* < 0.001). Compared to the reference, ratio 1 underestimated wedge height by −1 ± 2 mm (range: −4 to 3 mm; *p* = 0.024) and ratio 2 overestimated by 4 ± 2 mm (range: 1–8 mm; *p* = 0.005). The osteotomy depth method achieved a correction error within ±1° in 83% of cases (38/46), ratio 1 (50%, 23/46; *p* = 0.048) and ratio 2 (9%, 6/46; *p* < 0.001). Interrater ICC was 0.85 (95% confidence interval [CI]: 0.73–0.92) for PTS, 0.91 (95% CI: 0.83–0.95) for osteotomy depth and 0.89 (95% CI: 0.80–0.94) for wedge height.

**Conclusion:**

Fixed wedge height ratios resulted in a correction error greater than ±1° relative to the angular‐based method in up to 91% of infratubercle ACW‐HTOs. Wedge height calculated from osteotomy depth and correction angle provides superior planning precision.

**Level of Evidence:**

Level IV.

**Abbreviations**
ACL: anterior cruciate ligament
ACW‐HTO: anterior closing wedge high tibial osteotomy
mm: millimetre
PTS: posterior tibial slope
SD: standard deviation

## INTRODUCTION

Anterior closing wedge high tibial osteotomy (ACW‐HTO) has become an established procedure in revision anterior cruciate ligament reconstruction (ACLR) in patients with excessive posterior tibial slope (PTS) [4, 14, 22, 28, 35, 38]. Two surgical approaches are most commonly used: the infratubercle and supratubercle technique [15, 31]. The techniques differ in the osteotomy entry point at the anterior tibia and consequently affect the required wedge dimensions to achieve the desired angular correction [7, 8, 18, 20, 27, 29].

Preoperative planning for ACW‐HTO is typically performed on lateral knee radiographs to determine the degree of correction and the corresponding wedge height [13, 17, 30]. Multiple ratios for wedge height per degree of PTS correction have been proposed, ranging from 0.8 mm per degree to 1.67 mm per degree of correction [9, 18, 20, 29]. The application of fixed ratios assumes uniform tibial anatomy across patients, which may not reflect the considerable anatomical variability encountered in clinical practice [6, 18, 36].

Previous studies have demonstrated that measurements based on correction angle and osteotomy depth in medial opening wedge HTOs provide more accurate and reproducible corrections compared to wedge height alone [24, 32, 36]. The findings suggest that, rather than wedge height ratios, wedge height calculated from osteotomy depth may also be applied in closing wedge osteotomies. Accounting for individual tibial anatomy may be particularly important in ACW‐HTOs, given the considerable anatomical variation inherent to the proximal tibia.

The purpose of this study was to compare wedge heights resulting from different planning methods in infratubercle ACW‐HTO. The hypothesis was that utilising osteotomy depth and correction angle provides higher precision in planning ACW‐HTOs than applying wedge height ratios.

## MATERIALS AND METHODS

**Figure 1:**
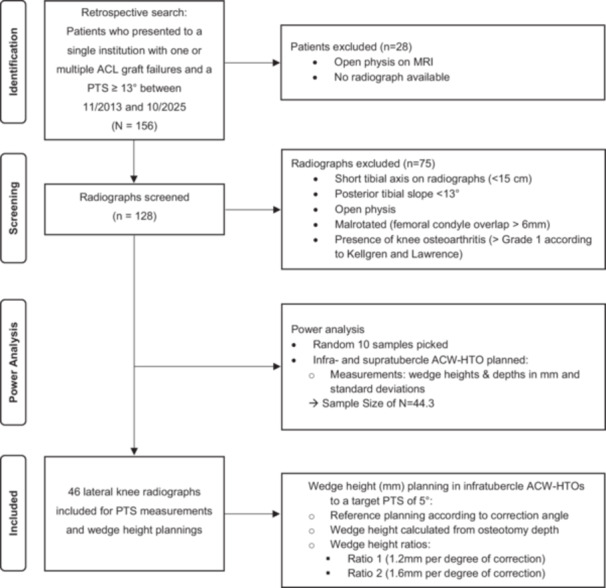
Flowchart of patient selection and radiographic analysis for medial posterior tibial slope (PTS) measurement and preoperative planning of wedge height and osteotomy depth (superior osteotomy cut) for infratubercle anterior closing wedge high tibial osteotomies (ACW‐HTOs) in millimetres (mm). Total number of subjects (*N*), number of subjects (*n*). ACL, anterior cruciate ligament.

This study was approved by the Institutional Review Board of the University of Pittsburgh (IRB# STUDY19030196). A retrospective chart review was conducted to identify patients who presented to the authors’ clinic between 01/2014 and 12/2022 with one or multiple ACL graft failures and a PTS ≥ 13°, who were considered for ACW‐HTO to correct excessive PTS. The PTS threshold of ≥13° was chosen based on published evidence linking elevated PTS with increased ACL graft failure risk [3, 10, 16, 17, 37]. Exclusion criteria were patients ≤18 years or >50 years or radiographic knee osteoarthritis ≥grade I [19] (Figure 1). Patients with a history of multiligamentous injuries, prior meniscus resections or meniscus transplants, a history of surgical cartilage procedures, history of tibial fractures or any history of potentially PTS‐altering surgical procedures or injuries, and poor quality of radiographs were excluded. Radiographs were defined as poor quality if the tibial shaft length was <15 cm, or if malrotation was present, defined as posterior femoral condyle overlap >6 mm or a posterior tibial condyle overlap >2 mm [11, 25, 34, 38]. Demographic data (age, sex and side) were recorded for all patients. All measurements were performed using the institutional picture archiving and communication system (PACS; Koninklijke Philips N.V.) and a calibration marker was used to account for variability in knee—detector distance.

### Radiographic measurements

**Figure 2:**
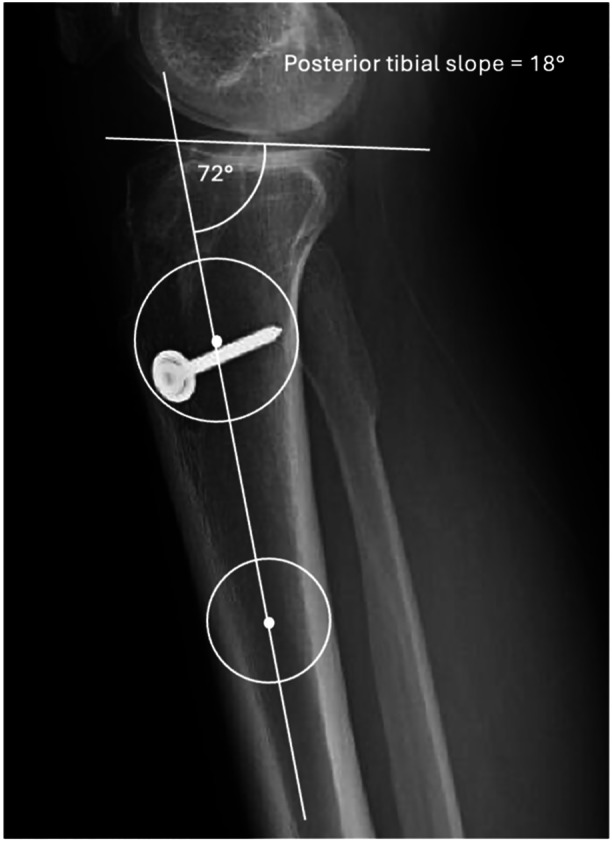
Lateral knee radiograph illustrating measurement of the medial posterior tibial slope (PTS). The medial PTS was defined as the angle between the anatomical tibial shaft axis and a line tangent to the medial tibial plateau. In this example, the measured angle was 72°. The PTS was calculated as 90° minus this angle, resulting in a medial PTS of 18°.

All measurements were performed by one medical student, one resident and one fellowship‐trained surgeon (R.P.V., R.E.B. and L.V.). One rater performed repeated measurements 6 weeks after the first round of measurements (R.P.V.). Measurements of the medial PTS were performed as previously described [5, 26]. The tibial axis was established by placing two circles along the tibial shaft, and the medial PTS was determined (Figure 2).

### Wedge height reference planning

**Figure 3:**
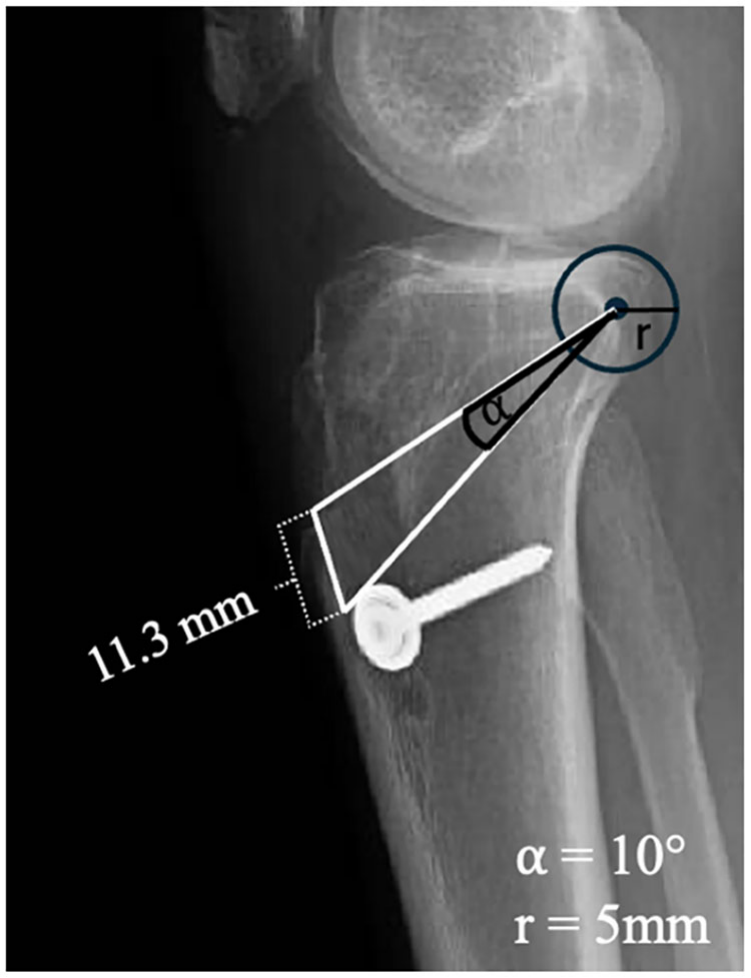
Infratubercle anterior closing wedge high tibial osteotomy (ACW‐HTO) (ACW‐HTO) reference planning: The superior osteotomy cut was drawn from the inferior insertion of the patellar tendon towards the hinge point. The inferior osteotomy cut was then drawn from the hinge point towards the anterior tibial cortex in the angle of the desired correction, defined as the correction angle (illustrated case: preoperative posterior tibial slope [PTS] = 15°, target PTS = 5°, correction angle *α *= 10°). Wedge height was measured as the distance between the intersections of both osteotomy cuts with the anterior tibial cortex (11.3 mm).

For planning purposes, a target PTS of 5° was used as this was suggested as the optimal correction target for ACW‐HTOs [2]. The hinge point was determined using a best‐fit circle on the posterior tibia (Figure 3) [18]. For the ACW HTO planning, the inferior insertion of the patella tendon was used as a reference for the entry point of the superior osteotomy line, which aimed towards the previously defined hinge point. The inferior osteotomy line was then projected in the desired correction angle. The correction angle is the angle formed by the superior and inferior cuts, to achieve a correction of the PTS to 5° (Figure 2). The correction angle was measured with the four‐point angle tool in the PACS system.

### Wedge height calculated from osteotomy depth

**Figure 4:**
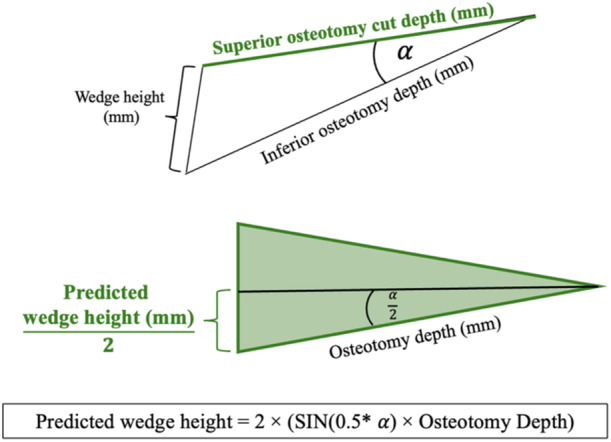
Determining the wedge height calculated from osteotomy depth and correction angle in an anterior closing‐wedge high tibial osteotomy (ACW‐HTO). In the case of ACW‐HTO, the depth of the superior cut (measured in millimetres) was used to define the osteotomy depth. The superior cut was chosen because during the infratubercle ACW‐HTO procedure, the superior cut is performed first. In closing wedge osteotomies, the wedge is created by two bone cuts that meet at a hinge point and form the correction angle. The approximated osteotomy wedge is composed of two congruent right‐angled triangles, with the hypotenuse representing the osteotomy depth and the angle α corresponding to half of the planned correction angle. The wedge height was calculated using the trigonometric sine (sin) function, according to the formula: wedge height = 2 × [sin(0.5 × α) × osteotomy depth].

The osteotomy depth was defined as the length of the superior cut of the infratubercle ACW‐HTO in millimetres (mm) [36]. The superior cut was selected because it is the first cut performed in infratubercle ACW‐HTOs and was further used to determine the wedge height. In closing‐wedge osteotomies, the bone wedge forms an acute, non‐isosceles triangle with unequal side lengths. In this model, the hypotenuse corresponds to the osteotomy depth, and the angle *α* represents half of the planned correction angle (Figure 4). The wedge height was calculated using basic trigonometric relationships, specifically the sine function.

### Wedge height ratios

**Figure 5:**
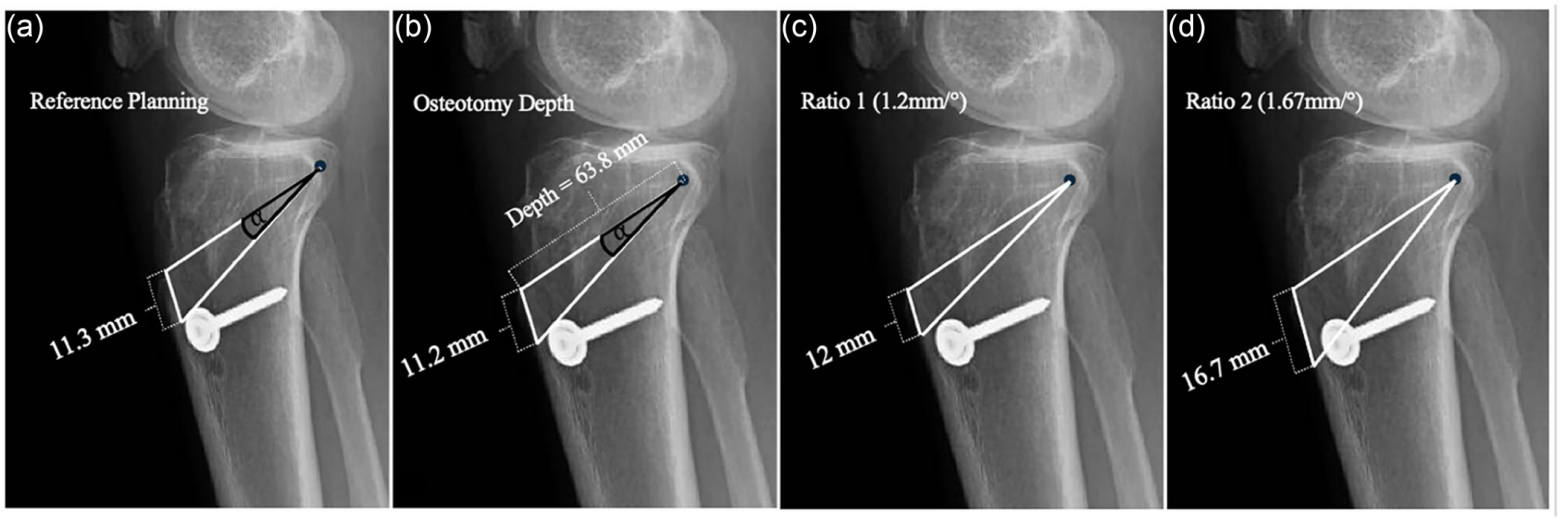
Lateral knee radiographs of the same patient demonstrating planning of anterior closing wedge high tibial osteotomies (ACW‐HTO). A posterior tibial slope (PTS) of 15° was planned to a target correction of PTS 5° (correction angle = 10°) according to different planning methods. The hinge point was defined by placing a circle with a 5‐millimetre (mm) radius (*r*) at the posterior curvature of the proximal tibia. The centre of this circle was used as the projected hinge point to ensure reproducibility across cases [16]. (a) Wedge height based on the correction angle (reference planning): the superior osteotomy cut was drawn from the inferior insertion of the patellar tendon towards the hinge point. The inferior osteotomy cut was then drawn from the hinge point towards the anterior tibial cortex using the correction angle (*α* = 10°). Wedge height was measured as the distance between the intersections of both osteotomy cuts with the anterior tibial cortex (11.3 mm). (b) Wedge height calculated from osteotomy depth: osteotomy depth (68.3 mm) and correction angle (10°) served as values for the trigonometric formula: wedge height = 2 × [sin(0.5 × α) × osteotomy depth]; (17.8 mm). (c) Ratio 1: Wedge height ratio of 1.2 mm per one degree of correction (wedge height = 12 mm). (d) Ratio 2: Wedge height ratio of 1.67 mm per one degree of correction (wedge height = 16.7 mm).

The wedge height ratio was defined as the specific amount of wedge height (mm) proposed for each degree of desired correction (mm/°). Wedge height ratios of 1.2 mm/° (ratio 1) and 1.67 mm/° (ratio 2) [9, 18, 30] were applied and included in the analysis. The wedge height was composed of the amount of degrees intended to correct targeting a PTS of 5°, times the wedge height ratio (ratio 1 and ratio 2, Figure 5).

### Statistical analysis

Descriptive statistics and tests for significance were calculated using SPSS version 27.0 (IBM Corp.) and presented in tables and figures. Continuous variables are reported as mean ± standard deviation. Differences in wedge height calculations between planning methods were analysed using a one‐way repeated‐measures analysis of variance (RM‐ANOVA), as all measurements were obtained from the same cases. The assumption of sphericity was assessed using Mauchly's test. In case of violation, Greenhouse–Geisser correction was applied to adjust the degrees of freedom. Effect sizes were reported as partial eta squared $\left(\eta_{p}^{2}\right)$. For targeted comparisons between the planning methods a pairwise post‐hoc analyses were performed using paired *t*‐tests. To account for multiple comparisons, Holm's correction was applied. Applying ratios in planning ACW‐HTO wedge heights yielded limited interindividual variability due to its deterministic calculation approach. The significance level was set at *α* = 0.05. Correction error was defined as the angular equivalent of the difference in planned wedge height between each method and the angular‐based reference, calculated using the inverse of the trigonometric formula described above. A correction error within ±1° was used to classify each case as within acceptable planning precision [23]. No osteotomies were performed or simulated; all correction errors represent mathematically planned deviations.

A priori power analysis was conducted using G*Power (version 3.1, Universität Düsseldorf, Germany) to determine the required sample size [12]. A random subset of 10 cases was used to estimate the variability of wedge height across subjects, yielding a standard deviation of 2.4 mm. This value reflects interindividual differences in wedge height. Assuming a minimum detectable difference of 1.0 mm [23], a two‐sided significance level of *α* = 0.05, and a power of 80%, the resulting standardised effect size was Cohen's *d* = 0.42, corresponding to a required sample size of 45.3 observations for paired comparisons. Therefore, a sample size of 46 cases was included. Intrarater reliability was assessed using the intraclass correlation coefficient (ICC) based on repeated measurements performed by a single rater (R.P.V.) at a 4‐month interval. Interrater reliability among three raters (R.P.V., R.E.B. and J.M.W.) was evaluated using the ICC and interpreted according to the criteria proposed by Koo and Li (<0.50 poor, 0.50–0.75 moderate, 0.75–0.90 good, >0.90 excellent) [21]. Intrarater ICC was 0.92 (95% confidence interval [CI]: 0.85–0.96). Interrater ICC was 0.85 (95% CI: 0.73–0.92) for PTS, 0.91 (95% CI: 0.83–0.95) for osteotomy depth and 0.89 (95% CI: 0.80–0.94) for wedge height.

## RESULTS

A total of 46 radiographs of 46 patients were included. The selection process is displayed in Figure 5. The mean age of patients was 30 ± 11 years (range: 18–50 years, 44% female). All measurements are presented in Table 1.

**Table 1:** Different planning methods to determine wedge heights in ACW‐HTO (*N* = 46).

	Mean	SD	Min	Max
Wedge height reference planning based on correction angle				
Wedge height (mm)	13	3	10	21
Wedge height calculated from osteotomy depth				
Wedge height (mm)	12	3	8	20
Delta wedge height (osteotomy depth vs. reference)	−2	1	−2	−1
Ratio 1 (Ratio 1.2 mm/°)				
Wedge height (mm)	12	2	10	17
Delta wedge height (Ratio 1 vs. reference)	−1	2	−4	3
Ratio 2 (Ratio 1.67 mm/°)				
Wedge height (mm)	17	3	13	23
Delta wedge height (Ratio 2 vs. reference)	4	2	1	8

Abbreviations: °, degrees; ACW‐HTO, anterior closing wedge high tibial osteotomy; max, maximum; min, minimum; mm, millimetre; SD, standard deviation.

### Comparison of wedge heights

**Figure 6:**
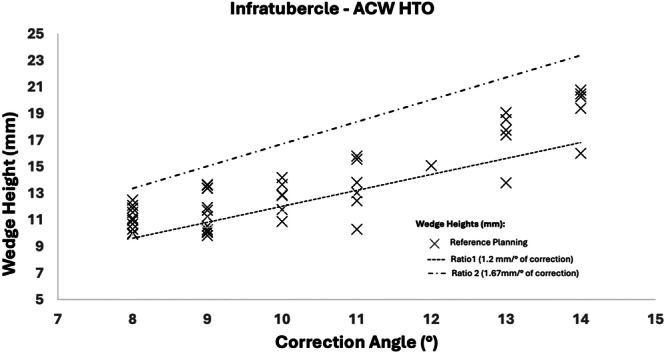
Graphical relationship between planned wedge heights measured in millimetres (mm) for anterior closing wedge high tibial osteotomy (ACW‐HTO). Wedge heights according to reference planning (×). Ratio 1 and C: The dashed lines indicate planning with wedge height ratios: Ratio 1 (1.2 mm/° of correction) and Ratio 2 (1.67 mm/° of correction).

**Figure 7:**
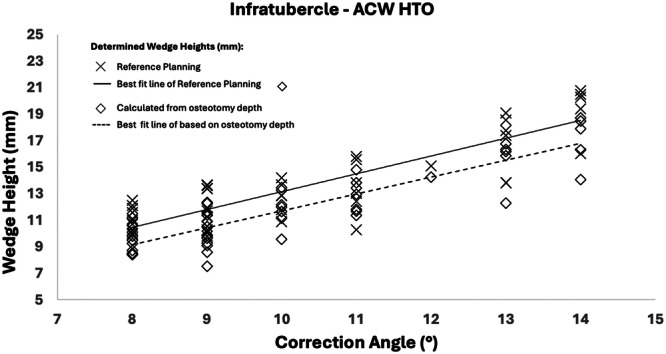
Graphical relationship between the reference planning of wedge heights measured in millimetres (mm) for anterior closing wedge high tibial osteotomy (×) and the osteotomy depth method; wedge heights calculated from osteotomy depth (). ACW‐HTO, anterior closing wedge high tibial osteotomy.

Comparison of ACW‐HTO wedge heights across planning methods (reference, osteotomy depth method, ratio 1 and ratio 2) demonstrated a significant overall difference of the applied calculation approach (repeated‐measures ANOVA with Greenhouse–Geisser correction; *p* < 0.001). When applying ratio 1 (ratio of 1.2 mm/° of correction), the mean wedge height was 12 ± 2 mm (range: 10–17 mm), and applying ratio 2 (1.67 mm/° of correction) yielded a mean wedge height of 17 ± 3 mm (range: 13–23 mm Table 1, Figure 6). The osteotomy depth method resulted in a mean wedge height of 12 ± 3 mm (range: 8–20 mm, Figure 7). Post‐hoc paired comparisons demonstrated that wedge heights obtained using planning methods ratio 1 and 2 differed significantly from the reference planning (all Holm‐adjusted *p* = 0.012).

### Planning precision of wedge heights

The osteotomy depth method achieved a correction error within ±1° in 83% of cases (38/46). In contrast, ratio 1 and ratio 2 showed lower predicted planning precision: ratio 1 resulted in 50% (23/46) of cases exceeding ±1° of the intended correction and was statistically significant from the reference planning (*p* = 0.048). Ratio 2 resulted in only 9% (6/46) of cases within ±1° of the intended correction, which was statistically significant compared to both the reference planning (*p* < 0.001) and the osteotomy depth method (*p* < 0.001). The relationship between correction angle, osteotomy depth and the resulting wedge height is summarised in Table 2.

**Table 2:** Wedge heights depending on the osteotomy depth (mm) and correction angle (°).

Correction angle (°)		Osteotomy depth (mm)							
		45	50	55	60	65	70	75	80
	7	5.5	6.1	6.7	7.3	7.9	8.5	9.2	9.8
	8	6.3	7.0	7.7	8.4	9.1	9.8	10.5	11.2
	9	7.1	7.8	8.6	9.4	10.2	11	11.8	12.6
	10	7.8	8.7	9.6	10.5	11.3	12.2	13.1	13.9
	11	8.6	9.6	10.5	11.5	12.5	13.4	14.4	15.3
	12	9.4	10.5	11.5	12.5	13.6	14.6	15.7	16.7
	13	10.2	11.3	12.5	13.6	14.7	15.8	17	18.1
	14	11	12.2	13.4	14.6	15.8	17.1	18.3	19.5
	15	11.7	13.1	14.4	15.7	17	18.3	19.6	20.9
		Wedge height in mm							

*Note*: Values were calculated using the formula wedge height = 2 × sin (0.5 × α) × osteotomy depth.

Abbreviation: mm, millimetre.

## DISCUSSION

The most important finding of the present study was that, in infratubercle ACW‐HTO, accurate planning was achieved using wedge height calculated from osteotomy depth relative to the angular‐based reference planning method. Planning accuracy was defined as a correction error within ±1°, a threshold achievable even with PSI‐based planning. Using this criterion, the osteotomy depth method achieved acceptable planning accuracy in 83% of cases (38/46), compared to 50% for ratio 1 and 9% for ratio 2. In contrast, relying on wedge height ratios resulted in substantial deviations from the angular‐based reference, highlighting their limited suitability for ACW‐HTO planning. Taken together, these findings challenge the continued use of wedge height ratios and support a shift towards geometry‐based, patient‐specific planning using wedge height calculated from osteotomy depth to account for individual tibial anatomy.

Multiple wedge height ratios have been proposed to translate a desired PTS correction into a millimetric wedge height [9, 18, 29]. A large clinical series of infratubercle ACW‐HTOs illustrated substantial variability, reporting a mean wedge height of 9.5 ± 1.8 mm with a mean PTS correction of 8.0 ± 1.7° (range 4.6–16.0°). The range of the reported wedge heights (range 6–19 mm) indicated the absence of a linear relationship between wedge height and angular correction [30]. When considering the range of correction error of the current study when applying a ratio of 1.2 mm/°of correction (−3° to 4°) this may result in considerable correction error in the individual case [29]. The current study showed that ratios resulted in over‐ or undercorrection of greater than 1° in up to 91% of cases when planning an infratubercle ACW‐HTO on a lateral knee radiograph.

Geometric considerations such as different entry‐ or hinge points in the proximal tibia explained the observed discrepancies in wedge heights [7, 20, 36]. The current study provides a tool for accounting for changes in osteotomy depth, such as from a more inferior entry point. Depending on the desired correction angle and the depth of the osteotomy, the resulting wedge height varies. In infratubercle ACW‐HTO, applying a wedge height ratio of 1.2 mm per degree of correction showed no statistically significant difference compared to angular‐based planning, and is attributed to the generally greater osteotomy depth in infratubercle ACW‐HTO. The further the osteotomy entry point is from the hinge point, the smaller the clinically relevant change in angular correction will occur when altering wedge heights. If planned with a wedge height ratio of 1.2 mm/° of correction, 50% of the cases were either over‐corrected or under‐corrected by more than one degree.

When performing ACW‐HTOs, there may be considerable variability in surgical execution. The variability in the entry point of the osteotomy, deviations in osteotomy length, and unintended shifts of the hinge point may affect the execution of the preoperative plan. When a wedge height ratio is used for preoperative planning, an additional source of potential variability is introduced. The current study offers a simple method to control the desired correction angle in ACW‐HTOs based on osteotomy depth. Furthermore, unlike wedge height ratios, the relationship between osteotomy depth and correction angle remains reliable despite the variability in proximal tibia morphology, which is frequently encountered clinically [1, 6, 33].

Several limitations must be acknowledged. This was a two‐dimensional radiographic study based on lateral knee radiographs, and both measurement error and intraoperative execution error may influence the achieved wedge height and final PTS. Nevertheless, the primary objective of this study was not to eliminate all sources of variability, but to compare wedge height ratios with wedge height calculated from osteotomy depth. Furthermore, although a calibration marker was used to standardise millimetre‐based measurements, variability in individual knee‐to‐detector distances may introduce minor measurement error. Another limitation of the current study is that two‐dimensional lateral radiographs cannot fully account for the mediolateral divergence of tibial plateau projections, highlighting the need for three‐dimensional planning approaches in future studies. The present study does not assess postoperative PTS outcomes. No osteotomies were performed or simulated; all comparisons are based on mathematically derived planned wedge heights. Clinical validation in a prospective surgical cohort with postoperative radiographic follow‐up is required to confirm the translation of this planning method to the achieved correction. The present data suggest that ACW‐HTO planning should move away from generalised ratios and towards patient‐specific planning using wedge height calculated from osteotomy depth. The study proposes a simple geometric approach using wedge height calculated from osteotomy depth and the desired correction angle.

## CONCLUSION

Fixed wedge height ratios resulted in a correction error greater than ±1° relative to the angular‐based method in up to 91% of infratubercle ACW‐HTOs. Wedge height calculated from osteotomy depth and correction angle provides superior planning precision.

## AUTHOR CONTRIBUTIONS

**Romed Peter Vieider**: Conceptualisation; methodology; writing—original draft preparation; writing—review and editing. **Julius Maria Watrinet**: Conceptualisation; methodology; writing—review and editing. **Robert Bilodeau**: Methodology; writing—original draft preparation; writing—review and editing. **Sahil Dadoo**: Writing—review and editing. **Luilly Vargas**: Writing—review and editing. **Luke Thomas Mattar**: Methodology; writing—review and editing. **Mahmut Enes Kayaalp**: Writing—review and editing. **Johnathan Daniel Hughes**: Writing—review and editing. **Volker Musahl**: Conceptualisation; writing—review and editing.

## CONFLICT OF INTEREST STATEMENT

Volker Musahl declares educational grants, consulting fees and speaking fees from Smith & Nephew plc, educational grants from Arthrex and DePuy/Synthes, is a board member of ISAKOS and deputy editor‐in‐chief of KSSTA. Johnathan Daniel Hughes: Associate Editor of KSSTA, paid consultant to Smith and Nephew, editorial board of Annals of Joint. Mahmut Enes Kayaalp: Deputy editor‐in‐chief of KSSTA, Co‐editor of Joint Diseases and Related Surgery, Member of ESSKA U*45 Committee. The remaining authors declare no conflicts of interest.

## ETHICS STATEMENT

This study was approved by the Institutional Review Board of the University of Pittsburgh (IRB# STUDY19030196).

## Data Availability

The data that support the findings of this study are available on request from the corresponding author. The data are not publicly available due to privacy or ethical restrictions.
